# Food Safety Threats: Molecular Surveillance, Antibiogram and Virulence Profiling of Biofilm Forming *Enterococcus faecalis* in Bangladeshi Restaurants

**DOI:** 10.1002/mbo3.70157

**Published:** 2025-11-11

**Authors:** Saad As Shadique, Farhana Binte Ferdous, Md. Nahid Ashraf, Sabrina Sultana Rimi, Mohosin Kabir, Md. Tanvir Rahman, Md. Shafiqul Islam

**Affiliations:** ^1^ Department of Microbiology and Hygiene, Faculty of Veterinary Science Bangladesh Agricultural University Mymensingh Bangladesh; ^2^ Interdisciplinary Institute for Food Security Bangladesh Agricultural University Mymensingh Bangladesh; ^3^ Department of Pathology, Faculty of Veterinary Science Bangladesh Agricultural University Mymensingh Bangladesh

**Keywords:** antimicrobial resistance, biofilm‐formation, *E. faecalis*, food safety, MDR, public health, virulence

## Abstract

*Enterococcus faecalis* (*E. faecalis*) is a notable public health bacterium since it can thrive on high‐touch surfaces in restaurants. This study aimed to isolate *E. faecalis*, conduct antibiogram to determine resistance patterns, explore the virulence profile and observe biofilm‐forming properties. A total of 90 samples were collected from BAU restaurants, including high‐touch surfaces and popular food items. Initial isolation employed culture‐based method followed by Gram's staining technique and biochemical tests. Molecular confirmation was achieved via polymerase chain reaction (PCR) targeting the *ddl*_*E. faecalis*_ gene specific for *E. faecalis*. Antibiogram was performed using the Disc Diffusion Test for commonly used antibiotics. Genotypic detection of antibiotic resistance and virulence profile were also explored by PCR. Lastly, the Congo Red (CR) test was done to examine the biofilm‐forming isolates. Results indicated a prevalence (30%) of *E. faecalis* in both food and surface samples, with higher contamination rates in crowded areas. Antibiogram revealed high resistance to Penicillin (100%) and moderate to low resistance towards Tetracycline, Ciprofloxacin, Erythromycin and Chloramphenicol. Shockingly, *bla*_TEM_ gene was found in 81.48% of isolates, and 18.51% were detected as multidrug‐resistant. We found a very high prevalence of the virulence genes *fsrA*, *fsrB*, *fsrC*, *gelE*, *pil*, *agg,* and *ace*. Finally, the CR test revealed 33.33% and 44.44% isolates as strong and intermediate biofilm formers respectively. This study reinforces the significance of routine surveillance in combating the spread of antimicrobial resistance through the food chain and the prospective use of *E. faecalis* as a contamination marker.

## Graphical abstract

Nowadays, researchers have found Enterococci in many food items. *E. faecalis* prevalence, antimicrobial resistance, pathogenicity, and biofilm‐forming capacity in food samples and food outlets in Bangladesh are little known. Microbiological conditions in food preparation sectors must be examined to ensure food safety and meet optimum criteria for high‐quality food production.

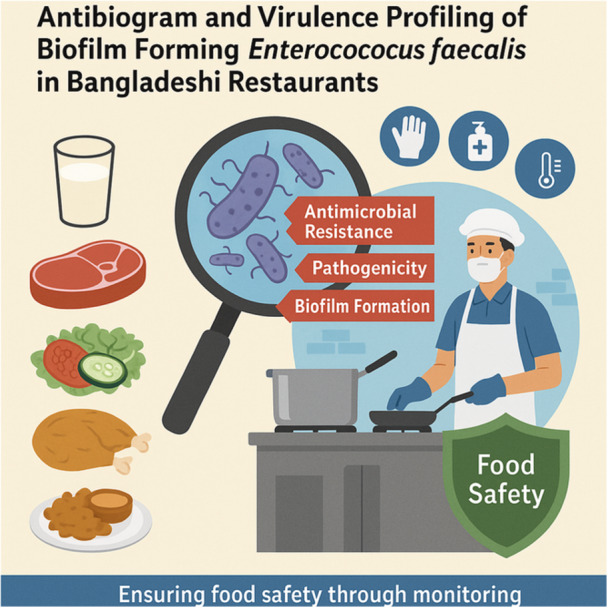

## 1 Introduction

There has been a significant shift in the food preferences of Bangladesh in recent years. Although the food typically handled, arranged, and sold at roadside eating establishments and other open sites is generally unhygienic, the tendency to eat out is on the rise (Higgs and Ruddock 2020). This alteration in behavior raised concerns about the quality of the food available, especially in terms of its hygienic and sanitary comfort (Ferreira et al. 2009; Coelho et al. 2010). Food contamination is primarily caused by the growth of objectionable microorganisms, which can result in the food being repugnant due to the deterioration or the health risks posed by pathogenic bacteria (Ferreira et al. 2009; Di Ciccio et al. 2015). A variety of foodborne pathogens that may lead to infections in humans have been reported by the Centers for Disease Control and Prevention (CDC) (Rahman et al. 2016). The proliferation of unwanted contaminating microorganisms can cause diseases in addition to harming the sensory and organoleptic qualities of food (Othman 2015).

Foodborne diseases (FBD) eventually result in economic losses by having a detrimental impact on the commerce and industries of the affected countries. Localized outbreaks of FBD may develop into a global threat (Bari and Yeasmin 2018). One of the primary outcomes of the collective feeding sector's lack of hygienic and sanitary control is the occurrence of these diseases, which are marked by biological, physical, and chemical hazards (Rahman et al. 2016). It is logical to assume that FBD are occasionally disseminated in hotels and restaurants through dishes, platters, and other kitchen equipment (Fawole and Oso 2007). The quality of cutlery, utensils, drinking cups, and dishes frequently determines the reputation of numerous hotels (Cracknel and Nobis 1989). On the other hand, the adhesion of microorganisms and the potential development of microbial biofilms may be facilitated by the existence of food residues and moisture on the preparation surface, which may increase the risk of cross‐contamination while making the cleansing process more difficult (Andrade 2008). Contaminated materials can directly expose pathogens to surfaces, or they can be indirectly exposed through microbiota in the air (Di Ciccio et al. 2015). We, as microbiologists, unequivocally demonstrate an association between the ingestion of adulterated food or beverages containing viruses, bacteria, or parasites (Linscott 2011). Additionally, Enterococci have been linked to contaminated outbreaks for their capability to transmit and disseminate resistance genes throughout the food chain and their presence in foods. The interaction between humans and Enterococci is intricate, and they are a significant group of bacteria (Oprea and Zervos 2007). Enterococci, which used to be thought of as microorganisms with little clinical impact, have now become a widespread opportunistic pathogen in humans (Teixeira and Merquior 2013). They are among the most common nosocomial pathogens that have the potential to cause fatal illnesses and infections (O'driscoll and Crank 2015). Enterococcal strains are the source of infections that can be transmitted from one individual to another or acquired through consuming contaminated food and water. These infections are derived from the patient's intestinal microbiota (Brilliantova et al. 2010). *Enterococcus faecalis* (*E. faecalis*) was associated with approximately 80% of these infections (Werner et al. 2008). Moreover, Enterococci have the ability to survive and expand in environments that are exceedingly hostile, thereby occupying an array of environmental niches. It seems that the gastrointestinal tract of humans and other animals is the principal natural habitat of these organisms (Oprea and Zervos 2007). For the purpose of applying a suitable antibiotic treatment, it is crucial to correctly determine a clinical isolate by medical and food microbiologists, as the susceptibility traits of different species are significantly distinct (Murray 1990; Morrison et al. 1997).

Antimicrobial resistance (AMR) is a serious widespread hazard that is getting increasingly concerning to the health of humans, animals, and their environment. This is the consequence of the persistence, dissemination, and emergence of multidrug‐resistant (MDR) bacteria, which is also referred to as “superbugs” (Davies and Davies 2010). AMR is likely to be caused by the overuse of antibiotics in both animals and humans (Ardakani et al. 2023). The feco‐oral route is the most prevalent mode of transmission at the community level, especially for Enterobacteriaceae pathogens which are resistant to antibiotics. This is typically the result of inappropriate sanitation (Aslam et al. 2018). The resistance of certain Enterococci to commonly used antibiotics is one of the remarkable virulence features that substantially enhance the pathogenicity of *Enterococcus* spp. by turning them into effective opportunistic microorganisms in nosocomial infections (Aslam et al. 2018; Landete et al. 2018; van Harten et al. 2017). Despite the fact that clinical enterococcal isolates contain a wider range of virulence determinants than Enterococci obtained from food, it is essential to exercise extra caution concerning AMR and virulence variables for Enterococci from food owing to the possibility of dissemination to the consumer (Ch'ng et al. 2019). Commensal bacteria, including Enterococci, have a capacity to carry the resistance characteristics to other bacterial genera as a consequence of their inherent gene transfer mechanism (McGowan‐Spicer et al. 2008).

Biofilm is a solitary microbial community that has established a permanent association with a surface, an interface, or each other. Enterococcal infections are substantially affected by biofilms. The tendency of bacteria to form biofilm enables them to thrive on an inert surface and provides the capacity to adhere to the host cells. The bacterium's ability to form biofilms and initiate infections depends on these genes' expression (McGowan et al. 2006).

In the recent days, researchers have detected the prevalence of Enterococci in a variety of foodstuffs. Nevertheless, there is a limited availability of data regarding the prevalence, antimicrobial susceptibility, virulence, and biofilm‐forming ability of *E. faecalis* in food samples and on the surface of food outlets in Bangladesh. It is crucial to examine the microbiological conditions of food preparation sectors to ensure the safety of food production and to stick to ideal standards for the production of high‐quality food items (Rana et al. 2023).

## 2 Materials and Methods

### 2.1 Sample Collection

A total 90 samples comprising restaurant swabs, fuchka, and raw chicken meat swab were collected during October, 2022–April,2023 from Bangladesh Agricultural University Campus areas (24.7363° N, 90.4245° E) by sterile cotton bud and kept in zip‐lock bags. The sampling areas were at several places and halls of BAU, including Jobbar's Moor (10); KR Market (10); Shes Moor (10); Fossil's Moor (10); Jamal Hossain Hall (5); Shahjalal Hall (5); Nazmul Ahsan Hall (5); Shamsul Haque Hall (5); Ashraful Haque Hall 5); Hossain Shaheed Suhrawardy Hall (5); Fozlul Haque Hall (5); Bangabandhu Sheikh Mujib Hall (5); Bangamata Sheikh Fazilatunnesa Mujib Hall (5); and Sultana Razia hall (5). Then, the samples were immediately transferred to the Department of Microbiology and Hygiene's laboratory at BAU in Mymensingh.

### 2.2 Isolation of *E. faecalis*

The samples were taken in nutrient broth and incubated overnight at 37°C and then streaked onto nutrient agar and selective media (*Enterococcus* agar base medium) (HiMedia, India) respectively to isolate *E. faecalis*. Additionally, the isolates were identified as *E. faecalis* through Gram's staining and recommended biochemical tests (Sugar fermentation test, Voges‐Proskauer test, Methyl red test, and catalase test).

### 2.3 Confirmation by PCR

Firstly, the genomic DNA of *E. faecalis* was extracted using boiling method, and afterward, PCR was conducted utilizing specific primers targeting *ddl*_*E. faecalis*_ gene. In brief, after culturing in nutrient broth at 37°C for 16–18 h, 1 ml of enriched broth was centrifuged at 5000 rpm for 5 min, and the supernatant was discarded. Later, the solution was prepared by adding 200 µl of PBS as the protocol outlined by Ferdous et al. (Doménech‐Sánchez et al. 2011). Then, the samples were subsequently boiled and chilled for 10 min and later centrifuged at 10,000 rpm for 10 min. Lastly, the supernatant was collected as a DNA template and frozen at −20°C for further use.

For the preparation of PCR products, 20 µl of PCR mixture was made by using nuclease‐free water (3 µl), master mixture (10 µl) (Promega, Madison, WI, USA), forward primer (1 µl), reverse primer (1 µl), and DNA template (5 µl) (Supporting Information Table S1).

### 2.4 Antibiotic Susceptibility Test

Disc diffusion test (DDT) was implemented to detect the antimicrobial susceptibility (Ferdous et al. 2023) by maintaining the Clinical and Laboratory Standards Institute's directives (Bauer et al. 1966). Initially, a sterile cotton swab was immersed into the suspension containing 4‐5 ml of nutrient broth and then incubated at 37°C for 24 h. After that, the bacterial inoculum was dispersed onto Mueller‐Hinton agar plates by setting them to 0.5 McFarland standard units (Clinical and Laboratory Standards Institute 2023). Finally, the selected antibiotic disks were positioned individually on Muller Hinton Agar plates. A total of eight antibiotics (Vancomycin/VA–30 μg, Ciprofloxacin/CIP–5 μg, Chloramphenicol/C–30 μg, Tetracycline/TE–30 μg, Penicillin/P–10 μg, Linezolid/LZD–30 μg, Nitrofurantoin/NIT–300 μg, and Erythromycin/E–15 μg) which are frequently prescribed against gram‐positive organisms in humans and animals were selected. Isolates that exhibited resistance to a minimum of three antimicrobial groups were categorized as MDR. The multiple antibiotic resistance (MAR) indices were calculated by dividing the number of resistant antibiotics by the total number of antibiotics tested (Clinical and Laboratory Standards Institute 2023).

### 2.5 Virulence Genes Detection

For the detection of virulence genes *fsrA*, *fsrB*, *fsrC*, *gelE*, *pil*, *agg,* and *ace, cyl* by PCR, DNA extraction followed by DNA amplification with specific primers (Supporting Information Table S2) of the respective virulence genes was done using the method mentioned earlier (Doménech‐Sánchez et al. 2011). After that, the amplified DNA products were made to run on a gel electrophoresis machine (Nippon Genetics, Tokyo, Japan) using 1.5% agarose (Invitrogen, Waltham, MA, USA). Then, the PCR products were submerged in ethidium bromide (HiMedia, Maharashtra, India) and checked for their expected amplicon sizes using an ultraviolet trans‐illuminator (Biometra, Göttingen, Germany). Additionally, the PCR‐positive controls included genomic DNA from *E. faecalis* that had previously shown positive results for the specific virulence genes (Clinical and Laboratory Standards Institute 2023). A 100‐bp DNA ladder (Promega, Madison, WI, USA) was used to detect the actual sizes of the bands.

### 2.6 Biofilm Forming Abilities

The biofilm‐forming ability of *E. faecalis* was detected phenotypically using the Congo red (CR) test, as described by Ferdous et al (Doménech‐Sánchez et al. 2011). In the CR assay, *E. faecalis* isolates were cultured on Congo red agar (CRA) plates to determine their biofilm‐forming abilities. To prepare CRA plates, 0.8 g of CR (HiMedia, Maharashtra, India) and 36 g of sucrose (HiMedia, Maharashtra, India) were mixed with 1000 mL of blood agar (HiMedia, Maharashtra, India). A sterility test was performed by incubating the mixture at 37°C overnight. Then, the cultured *E. faecalis* were steaked onto CRA agar and incubated similarly. After that, the distinct characteristics were assessed and examined (Ullah et al. 2024).

### 2.7 Statistical Analysis

The Statistical Package for Social Science (SPSS.v.25, IBM, Chicago, IL) was used for statistical analysis. Any variances in the virulence gene's frequencies were analyzed through descriptive analysis. The chi‐square test for relatedness was conducted to determine the association between phenotypic antibiotic resistance and virulence genes. An identical test (Z test for proportion) was also performed to analyze the association of biofilm formation with virulence genes and phenotypic antibiotic resistance, respectively. Additionally, a bivariate analysis was conducted to assess the potential correspondence between resistance patterns of any two antibiotics and to reveal the link between the virulence genes of *E. faecalis*. A p‐value below 0.05 was considered statistically significant.

## 3 Result

### 3.1 Occurrence of *E. faecalis*

**Figure 1:**
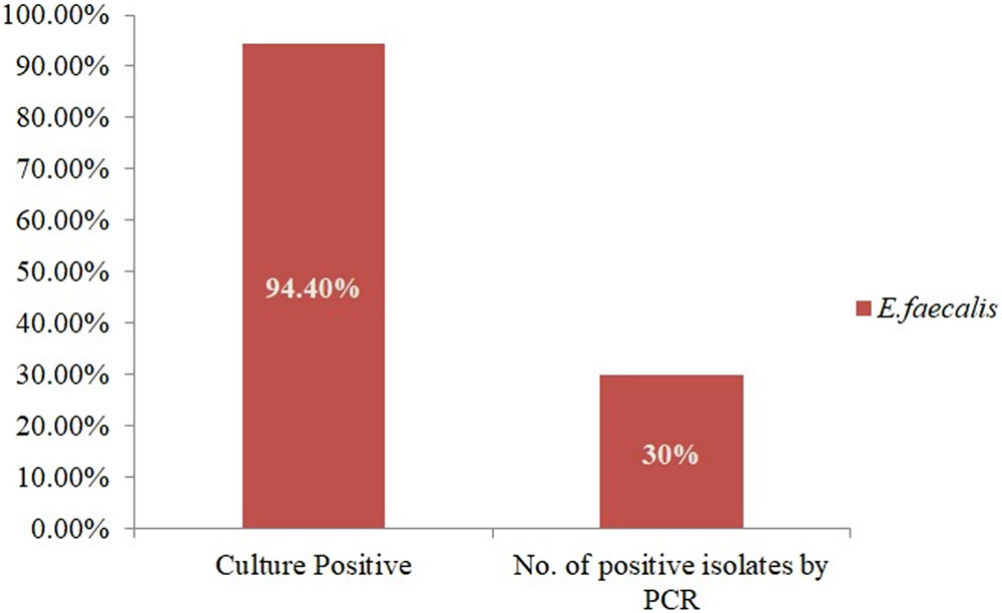
Overall occurrence of *E. faecalis* through culture and PCR.

Out of 90 samples, 94.4% (85/90; 95% CI: 87.64%–97.60%) were culture positive and 30% (27/90; 95% CI: 21.51%–40.127%) (Figure 1) isolates were found positive for *E. faecalis* by PCR that amplified by a band of 941 bp (Supporting Information Figure S1).

**Figure 2:**
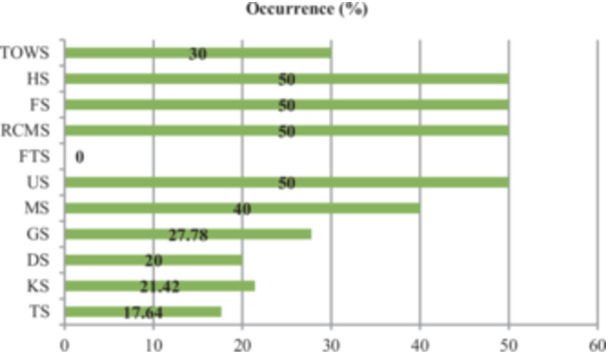
Occurrence of *E. faecalis* in different samples.

The occurrence varied depending on sample types, where the highest occurrence (50%, 95% CI: 25.38–74.62) was observed in Hand swab (HS), Fuchka Sample (FS), Raw Chicken Meat Swab (RCMS), and Utensils Swab (US). At the same time, 40%, 30%, 27.78%, 21.42%, 20%, and 17.64% *E. faecalis* were found in Menu swab (MS), Towel Swab (TOWS), Glass swab (GS), Kitchen Swab (KS), Door Swab (DS) and Table Swab (TS) respectively. Highest occurrence was found in KR market (50%, 5/10, 95% CI: 23.66–76.34) compared to other selected locations (Figure 2).

### 3.2 Antibiogram Profiles of *E. faecalis*

**Figure 3:**
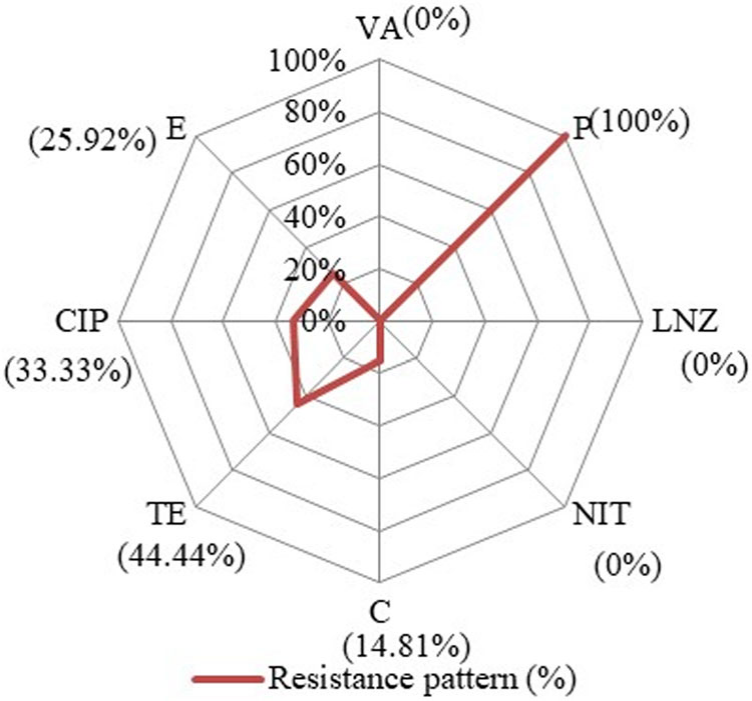
Resistance patterns of the isolates of *E. faecalis*.

In the disk diffusion method, all the 27 *E. faecalis* isolates were resistant to penicillin (27/27, 100%, 95% CI: 87.54–100) followed by Tetracycline (12/27, 44.44%, 95% CI: 27.58–62.68) Ciprofloxacin (9/27, 33.33%, 95% CI: 18.64–52.17), and Erythromycin (7/27, 25.92%, 95% CI 13.17–44.67). None of the Isolates were identified resistant towards Vancomycin, Linezolid and Nitrofurantoin (Figure 3). Genotypically, out of the 30 isolates, 22 (81.48%) were positive for *bla*_TEM_ (Supporting Information Figure S9). Moreover, 18.51% were found Multi‐Drug Resistant (MDR) (5/27, 18.51%, 95% CI: 8.2–36.7) and a total of eight antibiotic‐resistant patterns were detected, of which 3 patterns (pattern no. 1, 2, 3) exhibited MDR. The MAR indices varied from 0.125% to 0.5% and 92.59% (25/27, 95% CI: 76.63–98.68) of the isolates had more than 0.2 MAR index (Supporting Information Table S3). Additionally, in the chi‐square test, the presence of different virulent genes varied significantly among the penicillin‐resistant isolates (Supporting Information Table S4).

### 3.3 Occurrence of Virulence Genes in *E. faecalis*

In PCR, all the *E. faecalis* isolates harbored at least six investigated virulence genes. The highest number of *E. faecalis* isolates contained virulence genes *fsrA* (27/27, 100%, 95% CI: 87.54–100), *fsrB* (27/27, 100%, 95% CI: 87.54–100), *fsrC* (27/27, 100%, 95% CI: 87.54–100) and *gelE* (27/27, 100%, 95% CI: 87.54–100) followed by *pil* (26/27, 96.3%, 95% CI: 81.71–99.81), *agg* (24/27, 88.9%, 95% CI: 71.94–96.14) and *ace* (23/27, 85.2%, 95% CI: 67.52–94.08%). No *E. faecalis* isolates harbored the *cyl* gene. Bivariate analysis revealed a strong positive and significant correlation between *agg* and *pil* (Pearson correlation coefficient, ρ = 0.555, significance, *p* = 0.003) (Supporting Information Table S5). Moreover, the occurrence of virulence gene *agg* was significantly associated with different degrees of biofilm formation in *E. faecalis* isolates (Supporting Information Figures S2 **–** S8); (Supporting Information Table S6).

### 3.4 Biofilm Forming Ability of the Isolates

The biofilm assay revealed most of the *E. faecalis* as biofilm formers. The occurrence rate of intermediate biofilm‐forming isolates (12/27, 44.44%, 95% CI: 27.58–62.68) was higher than strong (9/27, 33.33%, 95% CI: 18.64%–52.17%) and weak (6/27, 22.23%, 95% CI: 10.60%–40.75%) biofilm‐forming *E. faecalis* isolates (Supporting Information Tables 7, 8).

## 4 Discussion

*Enterococcus* is not a typical foodborne pathogen; it is used as a probiotic to aid in the maturation of cheeses and sausages. However, its presence in food raises concerns about safety when ingested. Meanwhile, some strains are resistant to a wide variety of antibiotics. Enterococci are one of the leading causes of infections acquired in hospitals and cause illnesses, bacteremia, endocarditis, and other health conditions. Therefore, it matters that Enterococci are not carelessly announced as nonpathogenic bacteria, and the strains isolated from food should be cautiously assessed for all known virulence factors. *Enterococcus* spp. has been noticed as a potential hazard in foodstuffs by several scientists since the late 1990s. The design and information provided in these studies are subjected to several challenges. For example, most of the studies failed to convey an in‐depth and precise overview of the prevalence of *Enterococcus* spp. in food samples. They only assessed laboratory‐stored strains of *Enterococcus* spp. They were sourced from clinical or dietary samples to explore the phenotypic or genotypic traits. Since some publications recorded the detection rate of *Enterococcus* spp. in food samples, they primarily focused on ripened dairy products or fermented food items, within which Enterococci were, included as part of the starter cultures (Arciola et al. 2005). It is required to isolate and detect Enterococci from restaurant surfaces and food samples, as they signal sanitation gaps and serious public health hazards. Therefore, the current study isolated *E. faecalis* from restaurant surface samples to determine their prevalence. Additionally, antibiogram was conducted to uncover their resistance pattern, and their virulence profiles were explored by detecting various virulence genes. Finally, biofilm‐forming isolates were also checked in this study.

Several studies conducted in different countries have reported similar findings at Italy, the United States, Bangladesh, Argentina, and South Korea.… (McGowan‐Spicer et al. 2008; Kim et al. 2020a; Pesavento et al. 2014; Johnston and Jaykus 2004; Delpech et al. 2012) Although Tabashsum et al (Kim et al. 2020b). investigated various foodborne microorganisms in Bangladesh, including *Enterococcus* spp., they used a smaller sample size. They focused on various street foods such as Singara, Muri, Chatpati, Chetoi pitha, Sola, Jilapi, Jar water, Achar, Amra, Tehari, Vegetable Rolls, Sugarcane juice, sliced cucumber, and so on. Public health and sampling considerations led to an understanding that this study is being conducted for the first time in Bangladesh.

The present investigation revealed a discrepancy in the number of *E. faecalis* identified through the culture method compared to those detected by PCR. The variation between culture‐positive (85) and PCR‐positive (27) samples could result from differences between detection sensitivity and target specificity. PCR amplifies the ddl E. faecalis gene, hence verifying only genuine E. faecalis isolates, whereas culture methods might include additional Enterococcus species exhibiting same growth properties. PCR is considered more reliable, so this should be addressed. In this study, the isolates of *E. faecalis* were detected at a rate of 30%, which was lower than the percentages detected in raw meat (47.4%) and higher than the retail products (13.9%) reported by Pesavento et al (Kim et al. 2020a). At the same time, McGowan et al (McGowan‐Spicer et al. 2008). isolated a higher rate of *E. faecalis* (78.1%) from various meats and a reduced rate (19.8%) from fruits and vegetables. Furthermore, Kim et al (Delpech et al. 2012). conducted a study in South Korea to isolate *E. faecalis* along with other six *Enterococcus* species from meats and leafy vegetables. Their findings revealed the presence of a higher percentage of *E. faecalis* (40.4%) than the present study. The presence of *E. faecalis* declines in retail products due to processing techniques, including pasteurization, hygienic packaging, and rigorous regulatory controls, which limit contamination.

Raw products lack pathogen protection and are more susceptible. *Enterococcus faecalis* is more common in meat samples than fruits and vegetables because it comes from animals' gastrointestinal tracts. Subsequently, it raises the risk of contamination during slaughter and processing. Due to their lower nutrient content and less animal exposure, fruits and vegetables are less conducive to bacterial growth. Conversely, meat provides a nutrient‐rich environment for development. Poor handling, cross‐contamination, and storage facilities in meat processing may also contribute to the risk of *E. faecalis* contamination in meat over vegetables. Among the various sampling locations at BAU, the KR market was the most prevalent area for Enterococci contamination, with a 50% occurrence rate. We understand the likelihood of transmitting Enterococci increases with close human contact in congested places. Therefore, the crowdedness and hygienic practices in the KR market may be contributing factors to the higher occurrence of this organism than in other locations, such as Jobbar's Moor, Shes Moor, Fossil's Moor, and various gents and ladies halls of BAU.

Unsurprisingly, in recent decades, antibiotic resistance has become a significant concern for global health systems. As it is rising, and by 30 years, 10 million people will die annually from resistant bacteria infections, compared to 8.2 million from cancer. Resistant bacteria in food provide direct and indirect dangers to people. These bacteria may cause hard‐to‐treat FBD from improper cooking or cross‐contamination from other foods. Enterococci are linked to foodborne outbreaks because of their abundance in foods and their ability to spread resistance genes across the food chain. As antibiotic‐resistant Enterococci became more prevalent globally and sometimes worsened severe illnesses among people, there has been interest in uncovering their reservoirs and AMR genes (Oprea and Zervos 2007). Bacteria of the *Enterococcus* genus are utilized as sentinel organisms to track antibiotic resistance for Gram‐positive bacteria and as markers of animal fecal contamination into human food items (Tabashsum et al. 2013). Monitoring antibiotic resistance is of paramount importance to obtain information about the magnitude and patterns of the challenge and to prepare and provide subsequent follow‐up on the efficiency of any control measures that have been adopted (Tyson et al. 2018).

Previously several studies have explored the antibiotic resistance of *Enterococcus* spp. from different retail, ready‐to‐eat, raw foods and relevant equipment products (Kim et al. 2020a; Castaño‐Arriba et al. 2020; Jahan et al. 2013). Gaglio et al (Jahan et al. 2013). investigated Enterococci's virulence and AMR from equipment surfaces, raw materials, and traditional cheeses and reported nearly the same findings regarding resistance to Ciprofloxacin. However, the study of Chajęcka‐Wierzchowska and García‐Solache (Gaglio et al. 2016) revealed notably lower rates of resistance to P, TE, CIP, E, and C(3.1%, 16.9%, 0%, 18.5%, and 6.2% respectively) than that of the present study. This study found none of the isolates resistant to Vancomycin, Linezolid, and Nitrofurantoin, unlike the immediate prior study, which revealed slightly higher resistance rates (3.1%, 4.6%, and 7.7%, respectively). However, in the Tizi Ouzou region of Algeria, Titouche et al (Chajęcka‐Wierzchowska et al. 2020). Collected samples from various retail markets, butchers, dairy shops, and fast‐food outlets. They found 78.9% and 34.2% isolates resistant towards Tetracycline and Erythromycin, which were higher than those in our study but found significantly lower resistance for Penicillin and slightly lower resistance for Chloramphenicol. Their study was in line with ours as they found Vancomycin resistance. The frequent use or abuse of Penicillin in Bangladesh maximizes resistance, whereas other regions with tighter antibiotic control may observe more sensitive strains. The antimicrobial susceptibility testing carried out according standard protocols; however, the absence of a reference strain poses a limitation and underscores the necessity for its inclusion in future investigations to improve the validity of the data.

Beyond that, the *bla*_TEM_ gene was detected in 81.48% of the isolates. The management of infections arising from antimicrobial‐resistant organisms is not only challenging, but there is also an increased risk of severe illness and even mortality (Titouche et al. 2023)

This study identified 18.51% of isolates as MDR, which is alarming. Surprisingly, 92.59% of the isolates displayed MAR indices more than 0.2 that indicates the urge of controlling antibiotic usage and raising the public health concern (Salam et al. 2023). The ongoing existence and worldwide expansion of multi‐drug resistant bacteria is an aftermath of the failure of antibiotic treatments against “superbug” infections (Woh et al. 2023). The present research contradicts Ferdou*s* et al (Doménech‐Sánchez et al. 2011)., as no significant association was observed between biofilm formation and antibiotic resistance. The contradiction signifies that more research should be conducted to clarify these relationships fully. The occurrence and resistance pattern of *E. faecalis* may be impacted by multiple factors in multiple studies. The prevalence varied among studies due to variations in sample size, sample types, geographical locations, and environmental conditions. Variations also influence this in habitat, climate fluctuations and strain variability. Moreover, additional evidence of the resistance patterns would be recommended via detecting resistance genes for each selected antibiotic and the MIC test.

Even though Enterococci are only pathogenic under specific conditions, they are now one of the biggest drivers of human nosocomial infections. *E. faecalis* is more pathogenic due to its increased likelihood of containing human virulence factors (Lobanovska and Pilla 2017). Knowing the assortment and distribution of virulence genes in *Entercoccus* spp. is essential. They are isolated from distinct sources since they can be used to predict bacteria's pathogenicity in humans. Aggregation substances, cytolysin, enterococcal surface protein, gelatinase, and hyaluronidase, encoded by chromosomal genes, may contribute to the virulence of Enterococci. Absorption by intestinal epithelial cells and bacterial adhesion to other human cells are boosted by the aggregation substance encoded by *asa*1. The production of cytolysin exacerbates the severity of enterococcal diseases in humans. Enterococcal surface protein (*esp*) also boosts Enterococci's virulence, including general virulence, biofilm formation, colonization and endurance traits. Gelatinase and hyaluronidase, encoded by *gelE* and *hyl*, respectively, have been documented to aggravate infectious diseases, including pneumonia and endocarditis. Numerous studies have assessed the presence of these virulence factors in enterococcal isolates from foods (Arias et al. 2010; Hammad et al. 2015; Gomes et al. 2008; Barbosa et al. 2010). These studies were cross‐sectional, delivering a short overview of the prevalence, resistance, and virulence profile at a single point in time. To figure out the utility of policies aimed at ensuring safety and to review trends over time, continuous studies would be required. Our study observed a high prevalence of *fsrA*, *fsrB*, *fsrC*, *gelE*, *pil*, *agg*, and *ace* without the *cyl* gene. This finding aligns with the results reported by Rana et al. (McGowan et al. 2006). and Ullah et al. (Jahan and Holley 2014). Furthermore, a significant and positive correlation was found between the a*gg* and *pil* genes. Statistical analysis indicated significant variance in the occurrence of various virulence genes among the Penicillin‐resistant isolates.

This investigation specifically concentrated on *E. faecalis*, excluding other species of Enterococci. A more diverse set of *Enterococcus* strains and a larger sample size could be deployed to comprehensively understand the prevalence of *Enterococcus* spp. across various regions. The inclusion of some other potentially significant virulence factors (*esp*, *asa1*, *efaA*, and *hyl*) may provide greater insight into the pathogenicity of Enterococci. Furthermore, to look into the mechanisms of genetic transfer of virulence factors and resistant genes through the food chain, the samples of consumers can be collected from these areas from which the samples were taken.

More importantly, the pathogenesis of *E. faecalis* is linked to the bacteria's capacity to establish, adhere, invade, disrupt the host's defense mechanism, and develop biofilms. The bacteria's survival under hostile environmental conditions is facilitated by this fundamental characteristic (McGowan et al. 2006). Although the CRA test is not the most reliable method for evaluating biofilm development, the researchers selected it for its acceptable percentages of specificity and sensitivity (Doménech‐Sánchez et al. 2011). Our study found 33.33% of isolates as strong biofilm former. This study assessed phenotypic biofilm formation. However, the results would be more reliable if microtiter or other confirmatory analyzes were carried out. The process of biofilm formation is complex and affected by various factors. However, it can be partially credited to specific virulence factors, such as those associated with Enterococci's capacity to adhere and colonize the host (Doménech‐Sánchez et al. 2011). This study indicated that the virulence gene *agg* (aggregation substances), previously reported to enhance adherence and invasion of eukaryotic cells and promote biofilm formation (Ullah et al. 2023) was associated with varying degrees of biofilm formation. This finding was consistent with the findings of Soares et al (Anderson et al. 2016). and Anderson et al (Ullah et al. 2023)., who revealed a significant association between biofilm formation and the existence of virulence genes. The genus Enterococcus has been identified as one of the most alarming pathogens that can endanger life and health due to its high AMR and its ability to form biofilms. It is urgent to investigate and regulate the distribution of these microorganisms (Soares et al. 2014).

## 5 Conclusion

This study underscores the notable public health risk posed by biofilm‐forming, MDR Enterococcus faecalis in restaurants across Bangladesh. The detection of crucial virulence factors linked to biofilm formation, along with widespread antibiotic resistance and the presence of resistance genes like *bla*_TEM_, suggests a significant risk for ongoing environmental contamination and restricted therapeutic alternatives. The results highlight the paramount importance for regular microbial monitoring, strict adherence to hygiene protocols, and the implementation of public health education initiatives to mitigate the spread of resistant E. faecalis within the food supply chain.

## Author Contributions

**Saad As Shadique:** writing – original draft, visualization, software, methodology, investigation, formal analysis, data curation, conceptualization. **Farhana Binte Ferdous:** writing – review and editing, writing – original draft, visualization, software, methodology, investigation, formal analysis, data curation, conceptualization. **Md. Nahid Ashraf:** methodology, investigation. **Sabrina Sultana Rimi:** investigation. **Mohosin Kabir:** methodology, investigation. **Md. Tanvir Rahman:** writing – review and editing, supervision. **Md. Shafiqul Islam:** writing – review and editing, validation, supervision, resources, project administration, funding acquisition, conceptualization.

## Ethics Statement

The methodologies and related protocols used in this study were approved by the Institutional Ethical Committee (AWEEC/BAU/2024(2)/20(a)). In addition, verbal permission from the restaurant owner and waiter was taken during the sample collection.

## Conflicts of Interest

The authors declare no conflicts of interest.

## Supporting information

Supporting data to this article can be found in supplementary file.

**Supplementary Figure 1:** Molecular detection of ddl*E. faecalis* gene. Here M represents 100 bp DNA ladder, N‐negative control, P‐ positive control and 1‐9 (KS‐Kitchen Swab, FS‐Fuchka Sample, GS‐Glass swab, MS‐Menu swab, US‐Utensils Swab, RCMS‐Raw Chicken Meat Swab, FS‐Fuchka Sample, HS ‐ Hand swab, TOWS‐Towel Swab) represented PCR positive isolates of *E. faecalis.* **Supplementary Figure 2:** Molecular detection of *pil* gene. Here M represents 100 bp DNA ladder, N‐ negative control, P‐ positive control and 1 ‐ 8 (KS‐Kitchen Swab, GS‐Glass swab, MS‐Menu swab, US‐Utensils Swab, RCMS‐Raw Chicken Meat Swab, FS‐Fuchka Sample, HS ‐ Hand swab, TOWS‐Towel Swab) represented PCR positive for *pil* gene. **Supplementary Figure 3**: Molecular detection of *agg* gene. Here M represents 100 bp DNA ladder, N‐ negative control, P‐ positive control and 1 ‐ 8 (KS‐Kitchen Swab, GS‐Glass swab, MS‐Menu swab, US‐Utensils Swab, RCMS‐Raw Chicken Meat Swab, FS‐Fuchka Sample, HS ‐ Hand swab, TOWS‐Towel Swab) represented PCR positive for *agg* gene. **Supplementary Figure 4:** Molecular detection of *ace* gene. Here M represents 100 bp DNA ladder, N‐negative control, P‐ positive control and 1 ‐ 9 (KS‐Kitchen Swab, DS‐Door Swab, GS‐Glass swab, MS‐Menu swab, US‐Utensils Swab, RCMS‐Raw Chicken Meat Swab, FS‐Fuchka Sample, HS ‐ Hand swab, TOWS‐Towel Swab)represented PCR positive for *ace* gene. **Supplementary Figure 5:** Molecular detection of *fsrC* gene. Here M represents 100 bp DNA ladder, N‐negative control, P‐positive control and 1‐ 9 (KS‐Kitchen Swab, DS‐Door Swab, GS‐Glass swab, MS‐Menu swab, US‐Utensils Swab, RCMS‐Raw Chicken Meat Swab, FS‐Fuchka Sample, HS ‐ Hand swab, TOWS‐Towel Swab) represented PCR positive for *fsrC.* **Supplementary Figure 6:** Molecular detection of *fsrB* gene. Here M represents 100 bp DNA ladder, N‐negative control, P‐ positive control and 1‐ 8 (KS‐Kitchen Swab, GS‐Glass swab, MS‐Menu swab, US‐Utensils Swab, RCMS‐Raw Chicken Meat Swab, FS‐Fuchka Sample, HS ‐ Hand swab, TOWS‐Towel Swab) represented PCR positive for *fsrB* gene. **Supplementary Figure7:** Molecular detection of *fsrA* gene. Here M represents 100 bp DNA ladder, N‐negative control, PC‐ positive control and 1‐10 (TS‐Table Swab, KS‐Kitchen Swab, DS‐Door Swab, GS‐Glass swab, MS‐Menu swab, US‐Utensils Swab, RCMS‐Raw Chicken Meat Swab, FS‐Fuchka Sample, HS ‐ Hand swab, TOWS‐Towel Swab)represented PCR positive for *fsrA* gene. **Supplementary Figure 8:** Molecular detection of *gelE* gene. Here M represents 100 bp DNA ladder, N‐negative control, P‐ positive control and 1‐8 (KS‐Kitchen Swab, GS‐Glass swab, MS‐Menu swab, US‐Utensils Swab, RCMS‐Raw Chicken Meat Swab, FS‐Fuchka Sample, HS ‐ Hand swab, TOWS‐Towel Swab) represented PCR positive for *gelE* gene. **Supplementary Figure 9:** Molecular detection of *bla*TEM gene. Here M represents 100 bp DNA ladder, N‐negative control, P‐ positive control and 1‐9 (KS‐Kitchen Swab, DS‐Door Swab, GS‐Glass swab, MS‐Menu swab, US‐Utensils Swab, RCMS‐Raw Chicken Meat Swab, FS‐Fuchka Sample, HS ‐ Hand swab, TOWS‐Towel Swab) represented PCR positive isolates for *bla*TEM gene. **Supplementary Table 1:** List of primers with sequence. **Supplementary Table 2:** PCR condition against different primer sets for the amplification of species and virulence gene‐specific primers and *bla*TEM. **Supplementary Table 3:** Multidrug resistant *E. faecalis* with their resistance patterns. **Supplementary Table 4:** Association in the phenotypic antibiotic resistance and detection of virulence genes in *E. faecalis* (chi‐square test). **Supplementary Table 5:** Pearson correlation coefficient of virulence genes in *E. faecalis* isolates (bivariate analysis). **Supplementary Table 6:** Occurrence of Virulence genes in *E. faecalis* (Z test for proportion). **Supplementary Table 7:** Occurrence of biofilm forming isolates in *E. faecalis* (n=27). **Supplementary Table 8:** Association between the virulence genes and biofilm formation (Z test for proportion).

## Data Availability

All the data are available in the article and Supporting Information File S1.
