# Supplementary material for: Food Safety Threats: Molecular Surveillance, Antibiogram and Virulence Profiling of Biofilm Forming Enterococcus faecalis in Bangladeshi Restaurants
Source: Microbiologyopen. 2025 Nov 11;14(6):e70157. doi: 10.1002/mbo3.70157 (PMC12605974; doi:10.1002/mbo3.70157)
Supplement: Supplementary file 1 — Supplementary Figure 1: Molecular detection of ddlE. faecalis gene. Here M represents 100 bp DNA ladder, N‐negative control, P‐ positive control and 1‐9 (KS‐Kitchen Swab, FS‐Fuchka Sample, GS‐Glass swab, MS‐Menu swab, US‐Utensils Swab, RCMS‐Raw Chicken Meat Swab, FS‐Fuchka Sample, HS ‐ Hand swab, TOWS‐Towel Swab) represented PCR positive isolates of E. faecalis. Supplementary Figure 2: Molecular detection of pil gene. Here M represents 100 bp DNA ladder, N‐ negative control, P‐ positive control and 1 ‐ 8 (KS‐Kitchen Swab, GS‐Glass swab, MS‐Menu swab, US‐Utensils Swab, RCMS‐Raw Chicken Meat Swab, FS‐Fuchka Sample, HS ‐ Hand swab, TOWS‐Towel Swab) represented PCR positive for pil gene. Supplementary Figure 3: Molecular detection of agg gene. Here M represents 100 bp DNA ladder, N‐ negative control, P‐ positive control and 1 ‐ 8 (KS‐Kitchen Swab, GS‐Glass swab, MS‐Menu swab, US‐Utensils Swab, RCMS‐Raw Chicken Meat Swab, FS‐Fuchka Sample, HS ‐ Hand swab, TOWS‐Towel Swab) represented PCR positive for agg gene. Supplementary Figure 4: Molecular detection of ace gene. Here M represents 100 bp DNA ladder, N‐negative control, P‐ positive control and 1 ‐ 9 (KS‐Kitchen Swab, DS‐Door Swab, GS‐Glass swab, MS‐Menu swab, US‐Utensils Swab, RCMS‐Raw Chicken Meat Swab, FS‐Fuchka Sample, HS ‐ Hand swab, TOWS‐Towel Swab)represented PCR positive for ace gene. Supplementary Figure 5: Molecular detection of fsrC gene. Here M represents 100 bp DNA ladder, N‐negative control, P‐positive control and 1‐ 9 (KS‐Kitchen Swab, DS‐Door Swab, GS‐Glass swab, MS‐Menu swab, US‐Utensils Swab, RCMS‐Raw Chicken Meat Swab, FS‐Fuchka Sample, HS ‐ Hand swab, TOWS‐Towel Swab) represented PCR positive for fsrC. Supplementary Figure 6: Molecular detection of fsrB gene. Here M represents 100 bp DNA ladder, N‐negative control, P‐ positive control and 1‐ 8 (KS‐Kitchen Swab, GS‐Glass swab, MS‐Menu swab, US‐Utensils Swab, RCMS‐Raw Chicken Meat Swab, FS‐Fuchka Sample, HS ‐ Hand swab, TOWS‐Towel Swab) represented PCR [file MBO3-14-e70157-s001.docx]

**Supplementary Table**

**Supplementary Table 1: List of primers with sequence**

| **Target gene** | **Primer name** | **Sequence** | **Amplicon size (bp)** | **References** |
| --- | --- | --- | --- | --- |
| *ddl _E. faecalis_* | F | 5’-ATCAAGTACAGTTAGTCTT-3’ | 941 | Dutka Malen *et al.,* 1995 |
|  | R | 5’-ACGATTCAAAGCTAACTG-3’ |  |  |
| *bla*_TEM_ | F | 5’-CATTTCCGTGTCGCCCTTAT-3’ | 793 | Randall *et al.*, 2004 |
|  | R | 5’-TCCATAGTTGCCTGACTCCC-3’ |  |  |
| *agg* | F | TCTTGGACACGACCCATGAT | 413 | Hashem *et al.*, 2021 |
|  | R | AGAAAGAACATCACCACGAGC |  |  |
| *fsrA* | F | CGTTCCGTCTCTCATAGTTA | 474 |  |
|  | R | GCAGGATTTGAGGTTGCTAA |  |  |
| *fsrB* | F | TAATCTAGGCTTAGTTCCCAC | 428 |  |
|  | R | CTAAATGGCTCTGTCGTCTAG |  |  |
| *fsrC* | F | GTGTTTTTGATTTCGCCAGAGA | 716 |  |
|  | R | TATAACAATCCCCAACCGTG |  |  |
| *gelE* | F | GGTGAAGAAGTTACTCTGAC | 704 |  |
|  | R | GGTATTGAGTTATGAGGGGC |  |  |
| *ace* | F | GAATGACCGAGAACGATGGC | 615 |  |
|  | R | CTTGATGTTGGCCTGCTTCC |  |  |
| *pil* | F | GAAGAAACCAAAGCACCTAC | 620 |  |
|  | R | CTACCTAAGAAAAGAAACGCG |  |  |
| *cyl* | F | TGGCGGTATTTTTACTGGAG | 186 |  |
|  | R | TGAATCGCTCCATTTCTTC |  |  |

Legends, F = Forward, R = Reverse

**Supplementary Table 2: PCR condition against different primer sets for the amplification of species and virulence gene-specific primers and *bla*_TEM_**

| **Primer** | **Initial Denaturation** | | **Denaturation** | | **Annealing** | | **Extension** | | **Final extension** | |
| --- | --- | --- | --- | --- | --- | --- | --- | --- | --- | --- |
|  | **Tem** | **Time** | **Tem** | **Time** | **Tem** | **Time** | **Tem** | **Time** | **Tem** | **Time** |
| *ddl_E.faecalis_* | 95℃ | 5 m | 95℃ | 30 s | 54℃ | 30 s | 72℃ | 1m | 72℃ | 5 m |
| *bla*_TEM_ | 95℃ | 5m | 95℃ | 1m | 56℃ | 1m | 72℃ | 1m | 72℃ | 10m |
| *pil* | 95℃ | 5m | 95℃ | 1m | 53℃ | 45s | 72℃ | 1m | 72℃ | 5m |
| *ace* | 95℃ | 5m | 95℃ | 1m | 58℃ | 45s | 72℃ | 1m | 72℃ | 5m |
| *fsrA* | 95℃ | 5m | 95℃ | 1m | 53℃ | 45s | 72℃ | 1m | 72℃ | 5m |
| *fsrB* | 95℃ | 5m | 95℃ | 1m | 55℃ | 45s | 72℃ | 1m | 72℃ | 5m |
| *fsrC* | 95℃ | 5m | 95℃ | 1m | 54℃ | 45s | 72℃ | 1m | 72℃ | 5m |
| *gelE* | 95℃ | 5m | 95℃ | 1m | 52℃ | 45s | 72℃ | 1m | 72℃ | 5m |
| *agg* | 95℃ | 5m | 95℃ | 1m | 58℃ | 45s | 72℃ | 1m | 72℃ | 5m |
| *cyl* | 95℃ | 5m | 95℃ | 1m | 53℃ | 45s | 72℃ | 1m | 72℃ | 5m |

**Supplementary Table 3: Multidrug resistant *E. faecalis* with their resistance patterns**

| **No. of Pattern** | **Antibiotic Resistance Patterns** | **No. of Antibiotics (Classes)** | **No. of Isolates** | **MAR (Resistant antibiotics/ Total no. of antibiotics)** | **Overall MDR Isolates (%)** |
| --- | --- | --- | --- | --- | --- |
| 1 | P, TE, C, E | 4 (4) | 02 | 0.5 | 05 (18.51%) |
| 2 | P, CIP, TE | 3(3) | 02 | 0.375 |  |
| 3 | P, CIP, E | 3(3) | 01 | 0.375 |  |
| 4 | P, E | 2(2) | 06 | 0.25 |  |
| 5 | P, C | 2(2) | 02 | 0.25 |  |
| 6 | P, CIP | 2(2) | 05 | 0.25 |  |
| 7 | P, TE | 2(2) | 07 | 0.25 |  |
| 8 | P | 1(1) | 02 | 0.125 |  |

Legends, VA= Vancomycin (30 μg), CIP= Ciprofloxacin (5 μg), C= Chloramphenicol (30 μg), TE= Tetracycline (30 μg), P=Penicillin (10 μg), LZD=Linezolid (30 μg) NIT= Nitrofurantoin (300 μg), E=Erythromycin (15 μg)

**Supplementary Table 4: Association in the phenotypic antibiotic resistance and detection of virulence genes in *E. faecalis* (chi-square test)**

| **Resistant antibiotics** | **Virulence genes** | | | | | | | | ***p-value*** |
| --- | --- | --- | --- | --- | --- | --- | --- | --- | --- |
|  | *agg* (24) | *fsrA* (27) | *fsrB* (27) | *fsrC* (27) | *gelE* (27) | *ace* (23) | *pil* (26) | *cyl*(0) |  |
| P | 24(100^a^) | 27(100^a^) | 27(100^a^) | 27(100^a^) | 27(100^a^) | 23(100^a^) | 26(100^a^) | 0(0^b^) | 0.000 |
| C | 4(16.7^a^) | 4(14.8^a^) | 4(14.8^a^) | 4(14.8^a^) | 4(14.8^a^) | 4(17.4^a^) | 4(15.4^a^) | 0(0^a^) | 0.997 |
| TE | 10(41.7^a^) | 12(44.4^a^) | 12(44.4^a^) | 12(44.4^a^) | 12(44.4^a^) | 12(52.2^a^) | 11(42.3^a^) | 0(0^b^) | 0.192 |
| CIP | 9(37.5^a^) | 9(33.3^a^) | 9(33.3^a^) | 9(33.3^a^) | 9(33.3^a^) | 8(38.4^a^) | 9(34.6^a^) | 0(0^b^) | 0.692 |
| E | 6(25^a^) | 7(25.9^a^) | 7(25.9^a^) | 7(25.9^a^) | 7(25.9^a^) | 6(26.1^a^) | 7(26.9^a^) | 0(0^a^) | 0.931 |

Here, values with different superscripts differ significantly (P < 0.05) within the variable under assessment.

**Supplementary Table 5: Pearson correlation coefficient of virulence genes in *E. faecalis* isolates (bivariate analysis)**

| **Correlations** | |  |  |  |  |  |  |  |
| --- | --- | --- | --- | --- | --- | --- | --- | --- |
|  |  | ***Agg*** | ***fsrA*** | ***fsrB*** | ***fsrC*** | ***gelE*** | ***ace*** | ***Pil*** |
| ***agg*** | Pearson Correlation | 1 | .a | .a | .a | .a |  |  |
|  | Sig. (2-tailed) |  | . | . | . | . |  |  |
| ***fsrA*** | Pearson Correlation | .a | .a | .a | .a | .a |  |  |
|  | Sig. (2-tailed) | . |  | . | . | . |  |  |
| ***fsrB*** | Pearson Correlation | .a | .a | .a | .a | .a |  |  |
|  | Sig. (2-tailed) | . | . |  | . | . |  |  |
| ***fsrC*** | Pearson Correlation | .a | .a | .a | .a | .a |  |  |
|  | Sig. (2-tailed) | . | . | . |  | . |  |  |
| ***gelE*** | Pearson Correlation | .a | .a | .a | .a | .a |  |  |
|  | Sig. (2-tailed) | . | . | . | . |  |  |  |
| ***ace*** | Pearson Correlation | 0.184 | .a | .a | .a | .a | 1 |  |
|  | Sig. (2-tailed) | 0.357 | . | . | . | . |  |  |
| ***pil*** | Pearson Correlation | .555** | .a | .a | .a | .a | -0.082 | 1 |
|  | Sig. (2-tailed) | 0.003 | . | . | . | . | 0.685 |  |
| **cyl** | Pearson Correlation | .a | .a | .a | .a | .a | .a | .a |
|  | Sig. (2-tailed) | . | . | . | . | . | . | . |
| ** Correlation is significant at the 0.01 level (2-tailed). | | | | |  |  |  |  |
| a Cannot be computed because at least one of the variables is constant. | | | | | |  |  |  |

**Supplementary Table 6: Occurrence of Virulence genes in *E. faecalis* (Z test for proportion)**

| **Name of virulence gene** | **Occurrence (%)** | **SD** | **95% CI (%)** | **p-value** |
| --- | --- | --- | --- | --- |
| *agg* | 88.9^a^(24/27) | .320 | 71.94-96.14 | 0.000 |
| *fsrA* | 100^a^(27/27) | .000 | 87.54-100 |  |
| *fsrB* | 100^a^(27/27) | .000 | 87.54-100 |  |
| *fsrC* | 100^a^(27/27) | .000 | 87.54-100 |  |
| *gelE* | 100^a^(27/27) | .000 | 87.54-100 |  |
| *ace* | 85.2^a^(23/27) | .362 | 67.52-94.08 |  |
| *pil* | 96.3^a^(26/27) | .192 | 81.71-99.81 |  |
| *cyl* | 0^b^(0/27) | .000 | 0.00-12.45 |  |

Here, values with different superscripts differ significantly (P < 0.05) within the variable under assessment.

**Supplementary Table 7: Occurrence of biofilm forming isolates in *E. faecalis* (n=27)**

| **Name of Organism** | **Biofilm forming isolates** | **Occurrence of biofilm formers (%)** | **95% CI (%)** | ***p*-value** |
| --- | --- | --- | --- | --- |
| 1. *faecalis* | Strong | 33.33 | 18.64-52.17 | >0.05 |
|  | Intermediate | 44.44 | 27.58-62.68 |  |
|  | Non-biofilm former | 22.23 | 10.60-40.75 |  |

**Supplementary Table 8: Association between the virulence genes and biofilm formation (Z test for proportion)**

| **Virulence genes** | **Virulence genes in different degrees of biofilm formation** | | | ***p-value*** |
| --- | --- | --- | --- | --- |
|  | **No. (%) strong biofilm former (n = 9)** | **No. (%) intermediate biofilm former (n = 12)** | **No. (%) of non-biofilm former**  **(n = 6)** |  |
| *agg* | 9 (100^a^) | 12 (100^a^) | 3 (50^b^) | 0.003 |
| *fsrA* | 9(100^a^) | 12 (100^a^) | 6 (100^a^) | NA |
| *fsrB* | 9(100^a^) | 12 (100^a^) | 6 (100^a^) | NA |
| *fsrC* | 9(100^a^) | 12 (100^a^) | 6 (100^a^) | NA |
| *gelE* | 9(100^a^) | 12 (100^a^) | 6 (100^a^) | NA |
| *ace* | 8(88.9^a^) | 11 (91.7^a^) | 4 (66.7^a,b^) | 0.345 |
| *pil* | 9(100^a^) | 12 (100^a^) | 5 (83.3^a,b^) | 0.162 |
| *cyl* | 0(0^b^) | 0 (0^b^) | 0 (0^c^) | NA |

Here, values with different superscripts differ significantly (P < 0.05) within the variable under assessment.

**Supplementary Figure**

| 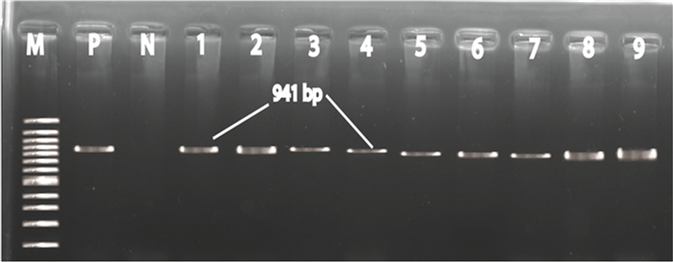 |
| --- |
| **Supplementary Figure 1:** Molecular detection of ddl*_E. faecalis_* gene. Here M represents 100 bp DNA ladder, N-negative control, P- positive control and 1-9 (KS-Kitchen Swab, FS-Fuchka Sample, GS-Glass swab, MS-Menu swab, US-Utensils Swab, RCMS-Raw Chicken Meat Swab, FS-Fuchka Sample, HS - Hand swab, TOWS-Towel Swab ) represented PCR positive isolates of *E. faecalis* |

| 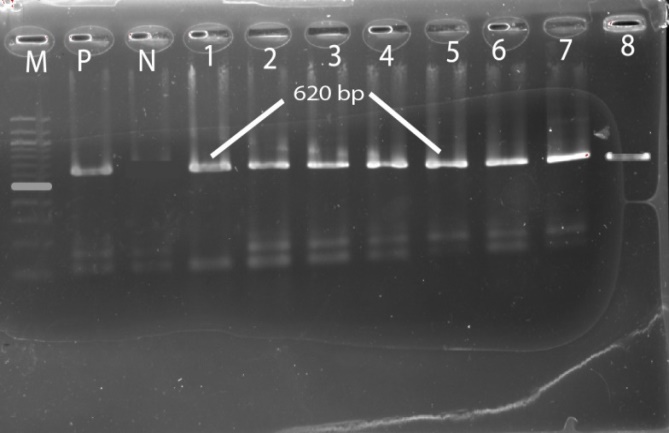 |
| --- |
| **Supplementary Figure 2:** Molecular detection of *pil* gene. Here M represents 100 bp DNA ladder, N- negative control, P- positive control and 1 - 8 (KS-Kitchen Swab, GS-Glass swab, MS-Menu swab, US-Utensils Swab, RCMS-Raw Chicken Meat Swab, FS-Fuchka Sample, HS - Hand swab, TOWS-Towel Swab) represented PCR positive for *pil* gene |

| 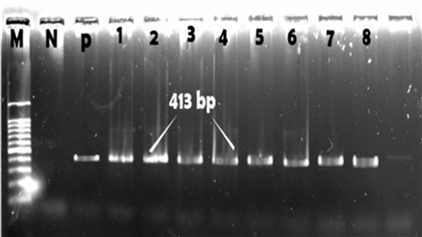 |
| --- |
| **Supplementary Figure 3**: Molecular detection of *agg* gene. Here M represents 100 bp DNA ladder, N- negative control, P- positive control and 1 - 8 (KS-Kitchen Swab, GS-Glass swab, MS-Menu swab, US-Utensils Swab, RCMS-Raw Chicken Meat Swab, FS-Fuchka Sample, HS - Hand swab, TOWS-Towel Swab) represented PCR positive for *agg* gene |

| 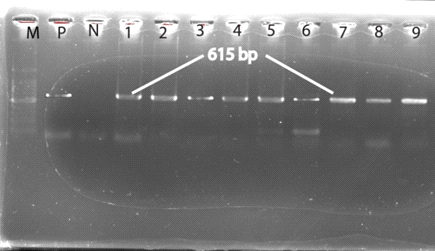 |
| --- |
| **Supplementary Figure 4:** Molecular detection of *ace* gene. Here M represents 100 bp DNA ladder, N-negative control, P- positive control and 1 - 9 (KS-Kitchen Swab, DS-Door Swab, GS-Glass swab, MS-Menu swab, US-Utensils Swab, RCMS-Raw Chicken Meat Swab, FS-Fuchka Sample, HS - Hand swab, TOWS-Towel Swab)represented PCR positive for *ace* gene |

| 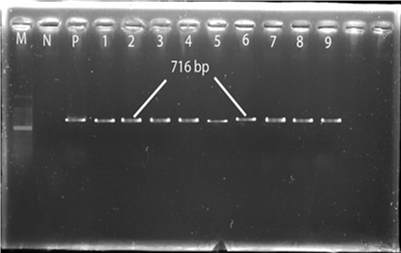 |
| --- |
| **Supplementary Figure 5:** Molecular detection of *fsrC* gene. Here M represents 100 bp DNA ladder, N-negative control, P-positive control and 1- 9 (KS-Kitchen Swab, DS-Door Swab, GS-Glass swab, MS-Menu swab, US-Utensils Swab, RCMS-Raw Chicken Meat Swab, FS-Fuchka Sample, HS - Hand swab, TOWS-Towel Swab) represented PCR positive for *fsrC* |

| 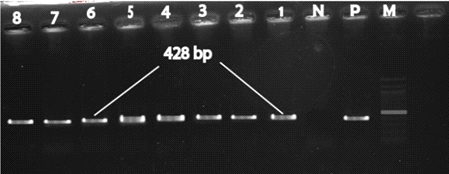 |
| --- |
| **Supplementary Figure 6:** Molecular detection of *fsrB* gene. Here M represents 100 bp DNA ladder, N-negative control, P- positive control and 1- 8 (KS-Kitchen Swab, GS-Glass swab, MS-Menu swab, US-Utensils Swab, RCMS-Raw Chicken Meat Swab, FS-Fuchka Sample, HS - Hand swab, TOWS-Towel Swab) represented PCR positive for *fsrB* gene |

| 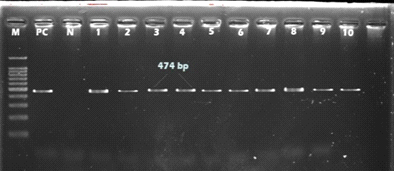 |
| --- |
| **Supplementary Figure7:** Molecular detection of *fsrA* gene. Here M represents 100 bp DNA ladder, N-negative control, PC- positive control and 1-10 (TS-Table Swab, KS-Kitchen Swab, DS-Door Swab, GS-Glass swab, MS-Menu swab, US-Utensils Swab, RCMS-Raw Chicken Meat Swab, FS-Fuchka Sample, HS - Hand swab, TOWS-Towel Swab)represented PCR positive for *fsrA* gene |

| 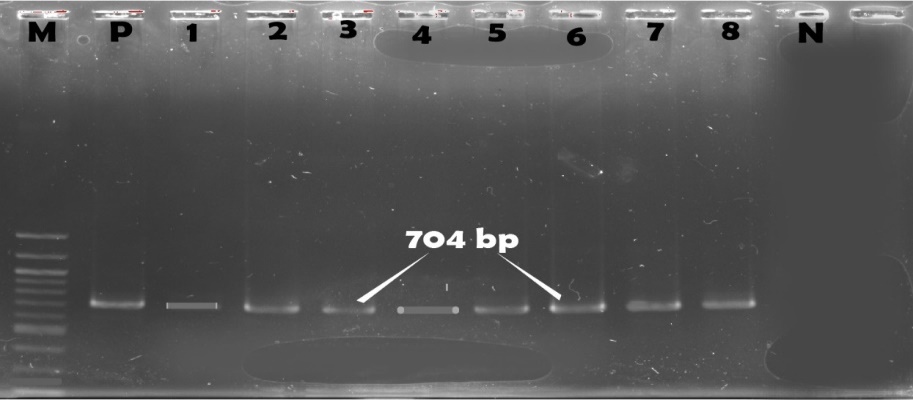 |
| --- |
| **Supplementary Figure 8:** Molecular detection of *gelE* gene. Here M represents 100 bp DNA ladder, N-negative control, P- positive control and 1-8 (KS-Kitchen Swab, GS-Glass swab, MS-Menu swab, US-Utensils Swab, RCMS-Raw Chicken Meat Swab, FS-Fuchka Sample, HS - Hand swab, TOWS-Towel Swab) represented PCR positive for *gelE* gene |

| 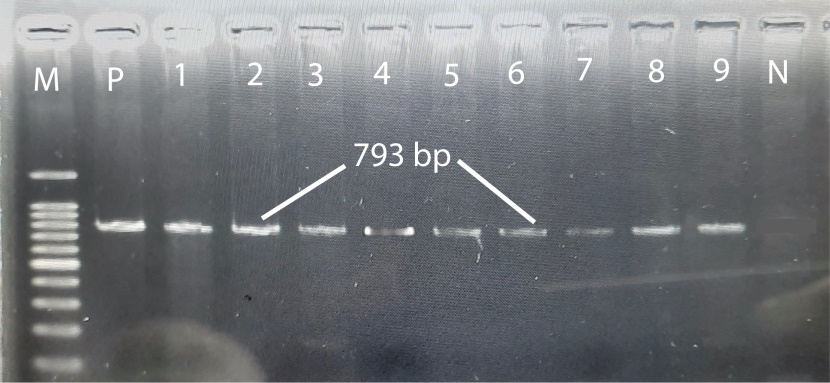 |
| --- |
| **Supplementary Figure 9:** Molecular detection of *bla*_TEM_ gene. Here M represents 100 bp DNA ladder, N-negative control, P- positive control and 1-9 (KS-Kitchen Swab, DS-Door Swab, GS-Glass swab, MS-Menu swab, US-Utensils Swab, RCMS-Raw Chicken Meat Swab, FS-Fuchka Sample, HS - Hand swab, TOWS-Towel Swab) represented PCR positive isolates for *bla*_TEM_ gene |
